# 

**Published:** 1896-02-08

**Authors:** 


### CONTENTS.

**A Prophylactic Clothes Philosophy** - - - - - 307

**The New Radiant Energy** - - - - - - - - 308

**The Treatment of Aneurism.** By A. PEARCE GOULD, F.R.C.S. - - - - - - - - - 309

**Modern Sociology.**—The Treatment of Criminals - - - 310

**Annotations.**—More Dangers from Tuberculosis—The Letsomian Lectures—The Extension of Rabies - - - - - 311

**Notes and News** - - - - - - - - 312

**Editor's Letter-box** - - - - - - - - - 321

**Medical Progress and Hospital Clinics—**

The Localisation of Tumours of the Spinal Cord amenable to Surgical Treatment. By E. PERCY PATON, M.D. - - - 313

PROGRESS IN MEDICINE.—Gout and Rheumatism - - - 315

PROGRESS IN SURGERY.—Brain Surgery (*continued*) - - - 316

PHYSICAL TREATMENT IN HEART DISEASE - - - - 318

NEW APPLIANCES AND THINGS MEDICAL - - - - - 318

**The Institutional Workshop—**

HOSPITAL SUNDAY FUND - - - - - - - - - 319

### EXTRA SUPPLEMENT.—THE NURSING MIRROR.

**News from the Nursing World.**—The Patients' Friends—Hampstead Nursing Association—Misleading Advertisements—A Loss to Mill Road Infirmary—More Work Wanted—Darlington District Nurses—Rainton District Nurse—District Nursing at Montrose—Nursing in Blairgowrie and Rattray—Proof Wanted—The late Miss Stewart—Dying and Dancing—Short Items - - - - - - - - - - clvii

**Lectures on Nursing.** IX.—Feeding - - - - - clix

**Lady Cyclists "On a Firm Social Basis"** - - - clx

**The Royal British Nurses' Association and its Difficulties** - - - - - - - - - - - clxi

**The Nurse's Co-operation** - - - - - - - - clx

**Our American Letter** - - - - - - - - - clx

**Workhouse Infirmary and Hospital Visiting** - - clxii

**Appointments** - - - - - - - - - - - clxii

**Nursing: Its Ideals and Realities** - - - - - clxiii

**The Present Position of Working Women** - - - cxliv

**Novelties for Nurses** - - - - - - - - - clxiv

**Notes and Queries** - - - - - - - - - - clxv

**Everybody's Opinion** - - - - - - - - - - clxv

**The Book World for Women and Nurses** - - - clxvi

**For Reading to the Sick** - - - - - - - - clxvi

